# Quasi-dendritic sulfonate-based organic small molecule for high-quality NIR-II bone-targeted imaging

**DOI:** 10.1186/s12951-023-01999-9

**Published:** 2023-07-19

**Authors:** Pengfei Chen, Fan Qu, Liuliang He, Mingfei Li, Pengfei Sun, Quli Fan, Chi Zhang, Daifeng Li

**Affiliations:** 1 Department of Orthopedics, The First Affiliated Hospital of Zhengzhou University, Zhengzhou, 450052 China; 2 State Key Laboratory of Organic Electronics and Information Displays and Institute of Advanced Materials (IAM), Jiangsu Key Laboratory for Biosensors, Nanjing University of Posts and Telecommunications, Nanjing, 210023 China

**Keywords:** NIR-II fluorescence imaging, Bone-targeted imaging, Sulfonate, Organic small molecule

## Abstract

The visualization of bone imaging in vivo is of great significance for the understanding of some bone-related diseases or physiological processes. Herein, a bone-targeted NIR-II small molecule (TTQF-SO_3_), which was modified with multiple sulfonate groups, was successfully fabricated for the second near-infrared (NIR-II) bone imaging. In vitro studies revealed that TTQF-SO_3_ showed high affinity for hydroxyapatite and excellent macrophage accumulation ability. In in vivo assays, TTQF-SO_3_ displayed high bone uptake ability and high NIR-II bone imaging quality, demonstrating the specific bone-targeting ability of the sulfonate-containing probe. In addition, the noninvasive NIR-II imaging detection in bone calcium loss was successfully verified in osteoporosis mice models. Moreover, the negative charge characteristic of TTQF-SO_3_ showed efficient lymphoid enrichment in living mice by intravenous injection. Overall, these warrant that our TTQF-SO_3_ is an optimal bone-targeted diagnostic agent for high-quality NIR-II imaging, highlighting its potential promise for clinical translation.

**Graphical Abstract:**

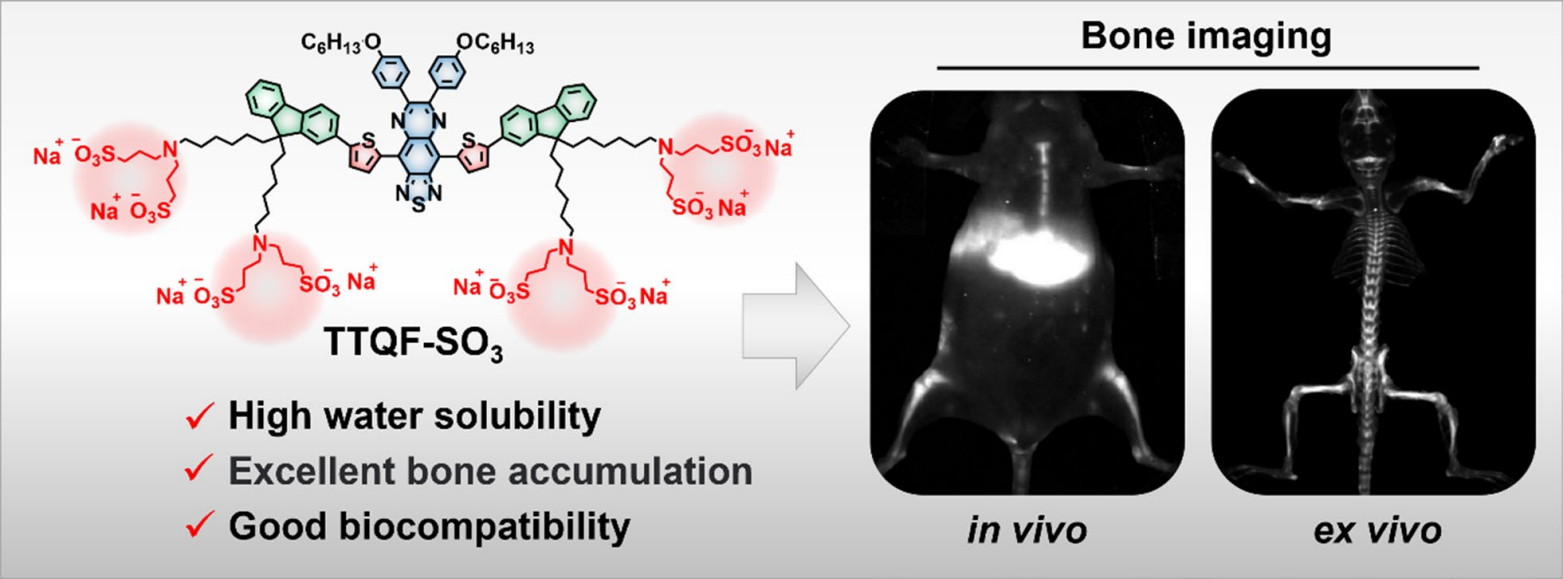

**Supplementary Information:**

The online version contains supplementary material available at 10.1186/s12951-023-01999-9.

## Introduction

Bone, as a dynamic tissue, is modeled and remodeled by a balance between osteoblast-induced mineralization and osteoclast-induced demineralization [1, 2]. The disruption of this balance is closely related to some diseases, such as osteoporosis, osteoarticular degeneration, and so on [3, 4]. Therefore, real-time visualization of in vivo bone imaging is of great significance for understanding some bone related diseases. However, clinical imaging of bone tissue is entirely based on X-ray or radiochemical methods, which is extremely limited. [5–7] By contrast, optical imaging has shown great potential for bone-imaging applications owing to its attractive properties such as nonhazardous, fast feedback, real-time imaging, and low cost [8–11]. Among them, fluorescence imaging (FI), especially the implementation of FI in the second near-infrared (NIR-II, 1000–1700 nm) region, is undoubtedly advantageous and promising in view of the deep tissue penetration, low autofluorescence, weak photon absorption/scattering, and high image resolution/sensitivity [12–15]. Therefore, developing bone-targeted NIR-II small-molecule-based fluorophores is highly desirable.

A common feature of most bone targeting agents is the strong affinity for Ca^2+^ salts. Taking bisphosphonates as the typical bone targeting groups, various bisphosphonate-based fluorescent probes have been extensively applied to bone imaging [16–18]. For example, several bisphosphonate-based cyanine derivatives have been reported for bone imaging [19–22]. Unfortunately, these molecules are limited to NIR-I (700–900 nm) region, which suffers from some shortcomings including poor tissue penetration, low resolution, and high background noise. Moreover, several reports have suggested that bisphosphonate-based fluorescent probes can induce osteoclast apoptosis, which may disrupt the physiological process that should be imaging [23]. More recently, Chen et al. were the first to demonstrate a bone-targeting NIR-II fluorescence probe using azide as bone-enriched group for the high-resolution osteoporosis imaging, but the highly reactive azide groups in the probe may have unknown potential toxicity [24]. Therefore, developing new NIR-II bone-targeting probes with high specificity, in vivo tolerance, excellent biocompatibility and the ability to monitor biological processes over extended periods of time is highly sought after.

Herein, we describe a new structural class of bone-targeting molecular probe with quasi-dendritic molecular structure, named TTQF-SO_3_. Specifically, the central scaffold of TTQF-SO_3_ is a donor–acceptor–donor (D–A–D)-based NIR-II fluorophore (TTQF). Through the synthetic chemistry method procedures specific numbers of peripheral sulfonate groups were attached to the central core (Additional file 1: Scheme S1). After systematic studies, including basic photophysical properties, calcium salt affinity, cell imaging, and in vitro imaging, TTQF-SO_3_ exhibits remarkable bone-seeking property, which is appropriate for further in vivo bone-targeted imaging. Under 808 nm laser radiation, the NIR-II fluorescence imaging (FI) of TTQF-SO_3_ can clearly observe the skeletal systems of healthy mice. In addition, the noninvasive NIR-II imaging detection in bone calcium loss was successfully verified in osteoporosis mice models. Notably, TTQF-SO_3_ can provide real-time and long-term observation of the entire skeletal system. Moreover, the negative charge characteristic of TTQF-SO_3_ showed efficient lymphoid enrichment in living mice by intravenous injection. Overall, TTQF-SO_3_ is an optimal bone-targeted diagnostic agent for high-quality NIR-II imaging with potential promise for clinical translation.

## Results and discussion

**Fig. 1:**
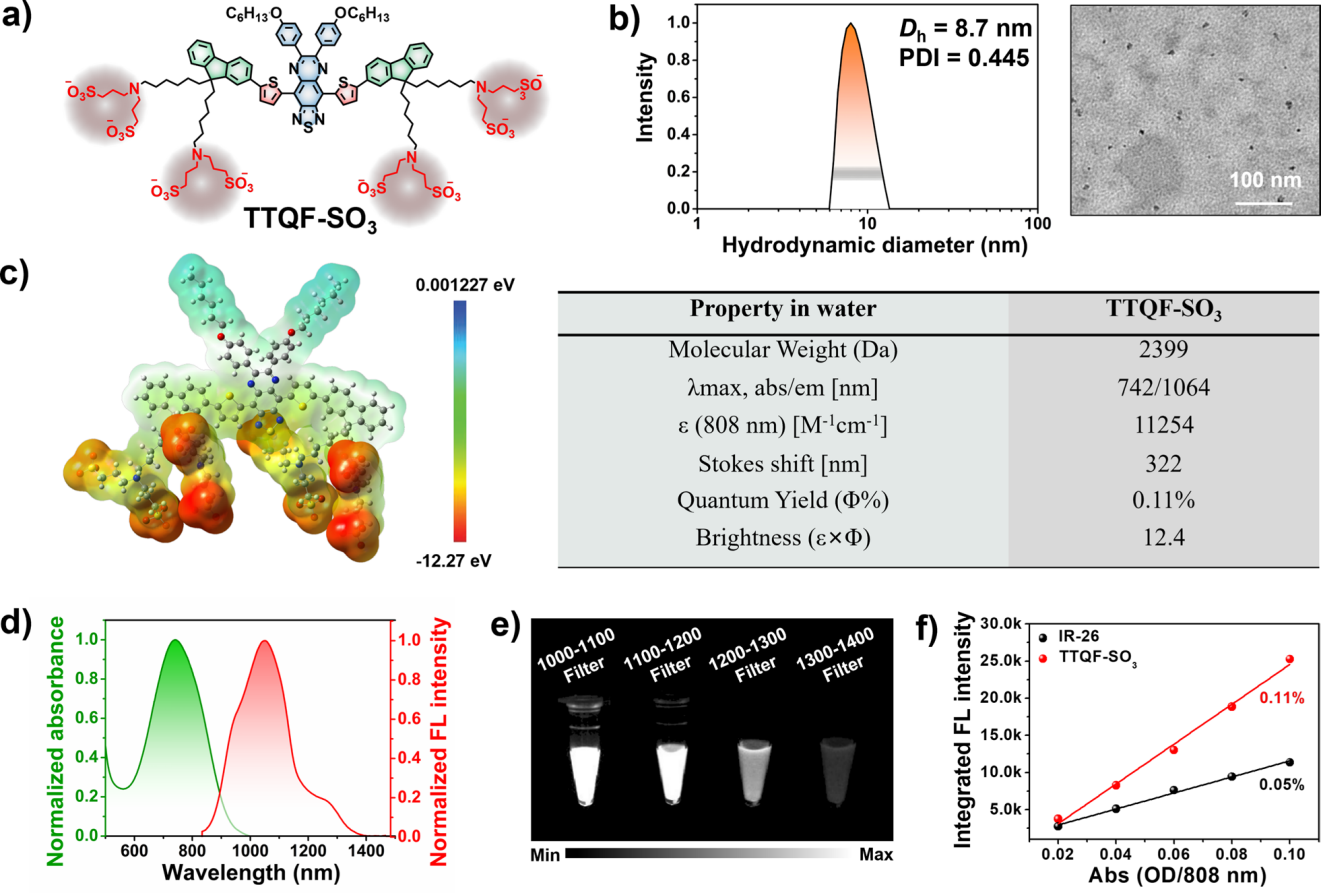
**a** Chemical structure of TTQF-SO_3_. **b** DLS and TEM image of TTQF-SO_3_ in water. **c** Physicochemical properties of TTQF-SO_3_. **d** Normalized absorption and emission spectrum of TTQF-SO_3_ in water. **e** NIR-II signals of TTQF-SO_3_ obtained with different filters at the same concentration (0.1 mg mL^−1^) in water. **f** The integrated fluorescence spectra of TTQF-SO_3_ and its versus different absorbance at 808 nm (IR-26 as reference, QY = 0.05% in dichloroethane)

The chemical structure of TTQF-SO_3_ is shown in Fig. 1a, and its detailed synthetic route is displayed in Additional file 1: Scheme S1. First, the D–A–D-based NIR-II dye TTQF-NH_2_ with functional amino groups was achieved from our previously reported method. Subsequently, TTQF-SO_3_ was obtained after sulfonation of the intermediate TTQF-NH_2_ with excess 1,3-propanesultone. The target TTQF-SO_3_ compound was thoroughly characterized by NMR spectroscopy and MALDI-TOF–MS analysis (Additional file 1: Figs. S1, S2). The eight terminal charged side chains of TTQF-SO_3_ enable a solubility in aqueous media greater than 100 mg mL^−1^, such that TTQF-SO_3_ dissolves into water directly without the assistance of any organic solvents (like dimethyl sulfoxide or alcohols) or encapsulation poly(ethylene glycol) coatings). Dynamic light scattering and transmission electron microscopy results revealed that TTQF-SO_3_ can self-assemble to form ultra-small dots with 8.7 nm average size (Fig. 1b). Zeta potential measurement revealed a negative surface charge (− 14.3 mV) for TTQF-SO_3_ in PBS (Additional file 1: Fig. S3a). Furthermore, TTQF-SO_3_ kept a good colloidal stability either in phosphate-buffered saline (PBS) and dulbecco’s modified eagle medium (DMEM) within 14 days (Additional file 1: Fig. S3b). Some other key properties of TTQF-SO_3_ in water media is shown in Fig. 1c. The absorption and fluorescence emission spectra of TTQF-SO_3_ were measured. The characteristic absorption and emission peaks were observed at around 742 nm and 1064 nm, respectively, with a significant stokes shift of 322 nm (Fig. 1d). The molar extinction coefficient of TTQF-SO_3_ at 808 nm was determined to be 11,254 M^−1^ cm^−1^ (Additional file 1: Fig. S4). The NIR-II signals of TTQF-SO_3_ were compared using various long-pass (LP) filters (1000–1400 nm) at the same concentration in water media, and found that the fluorescence signals can be clearly observed with each filter, even with the 1300–1400 nm LP filter (Fig. 1e). The quantum yield (QY) of TTQF-SO_3_ was determined to be 0.11%, using IR26 as a reference (QY = 0.05% in DCE, Fig. 1f and Additional file 1: Fig. S5). Moreover, the photostability of the TTQF-SO_3_ was much superior to that of IR1061, under continuous laser irradiation (Additional file 1: Fig. S6). All these results can be sufficient to support TTQF-SO_3_ for NIR-II medical imaging possibilities.

**Fig. 2:**
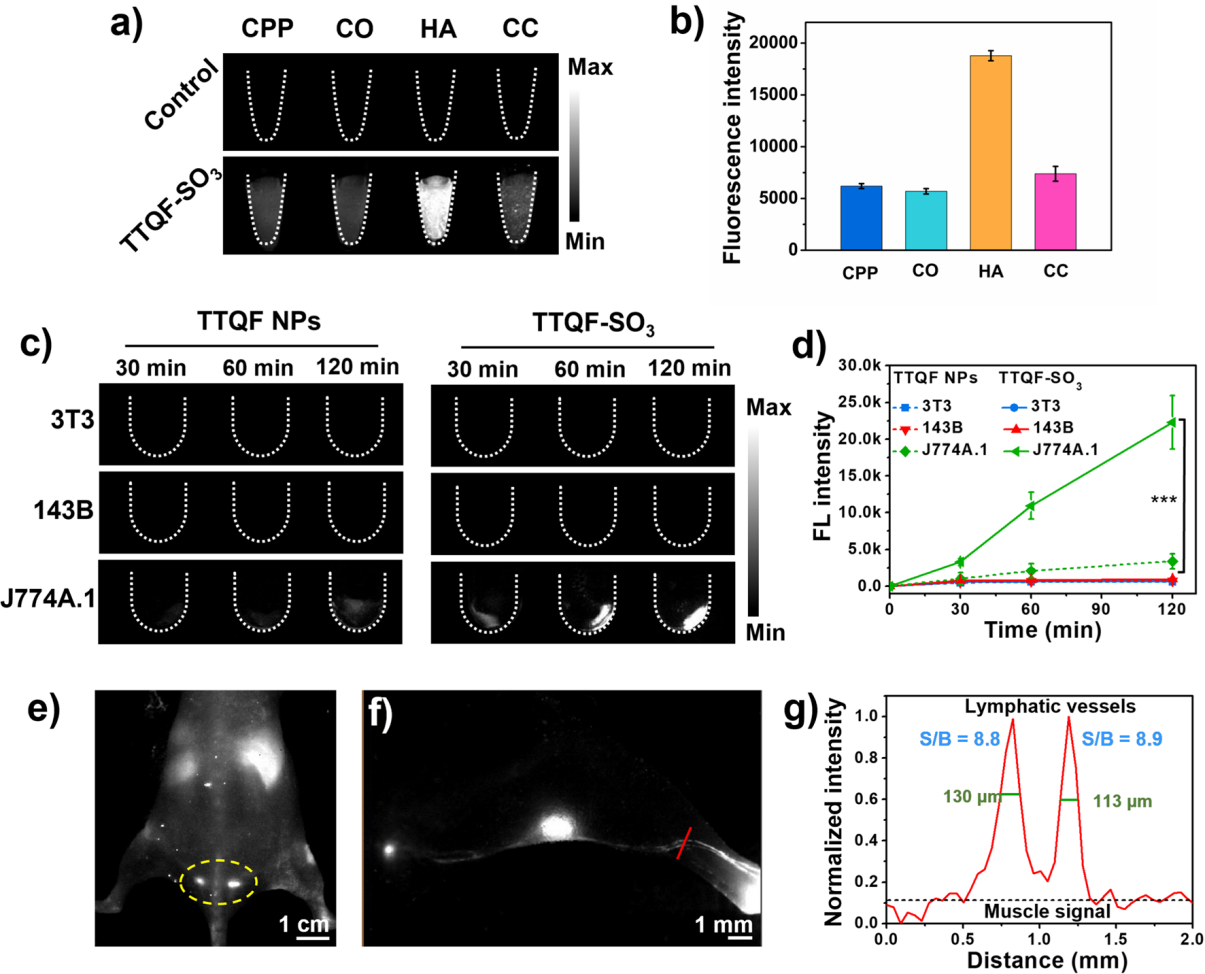
**a** Calcium-binding NIR-II fluorescence image (NIR-II FI) of TTQF-SO_3_. Imaging parameters: 1300 filter, exposure 500 ms. **b** Corresponding quantitative NIR-II fluorescent signals of TTQF-SO_3_. **c** NIR-II FI of different cells (3T3, 143B, and J774A.1) treated with TTQF NPs and TTQF-SO_3_ at different time points. Imaging parameters: 1300 filter, exposure 3000 ms. **d** Corresponding quantitative NIR-II fluorescent signals of TTQF NPs and TTQF-SO_3_ bound cells. **e** NIR-II FI of TTQF-SO_3_ in mice after intravenous injection. Imaging parameters: 1300 filter, exposure 3000 ms. **f** NIR-II FI of lymphatic vessels after footpad injection of TTQF-SO_3_ at 60 min. **g** Cross-sectional signal profile of lymphatic vessels in the selected region. Error bars, mean ± SD (n = 3). *p < 0.05, **p < 0.01, ***p < 0.001

The affinity binding ability to bone tissue minerals is usually an important criterion for evaluating bone-targeted imaging. To verify whether in vitro binding affinity of TTQF-SO_3_ toward inorganic calcium salt, different calcium-binding tests were investigated, including calcium pyrophosphate (CPP), calcium oxalate (CO), hydroxyapatite (HA), and calcium carbonate (CC). As shown in Fig. 2a, TTQF-SO_3_ demonstrated strong affinity for HA compared to other calcium salts, and its fluorescence intensity was about 3-folds than that of other calcium salts (Fig. 2b), which can be attributed to the hydroxyl group of HA contributes to form a strong hydrogen bond with the sulfonyl anion of the fluorophore. [21, 25] Since HA is a major component of bone, we expected that TTQF-SO_3_ would have a good bone-targeted ability in vivo.

Motivated by the above promising results, we conducted further tests at the cellular level. As is well known, the interaction between nanomaterials and cells mainly includes electrostatic interaction, water transport interaction, hydrogen bond interaction, π–π stacking and steric hindrance [26]. Among them, the surface charge properties of nanoparticles not only have a great effect on blood circulation, but also on cell uptake [27]. Therefore, three cell lines associated with bone metabolism, including embryonic fibroblast selection (3T3), osteosarcoma (143B), and mouse mononuclear macrophages (J774A.1), were selected for cell uptake studies [28]. The non-functionalized precursor TTQF nanoparticles (NPs) was prepared as a control group by co-deposition with the amphiphilic Pluronic F-127. Detailed characteristics of TTQF NPs were listed in Additional file 1: Fig. S7. The three kinds of cells were incubated with TTQF-SO_3_ and TTQF NPs, respectively for 30, 60, and 120 min, and their fluorescence signals were monitored by NIR-II imager. As shown in Fig. 2c, there was a significant difference in the fluorescence intensity of the two probes with macrophages at 120 min (*P* < 0.001). In contrast, the uptake of the two probes in 3T3 and 143B cells was nearly no difference. More accurately, these two probes are not easy to be absorbed by 3T3 and 143B cells in a short time. Considering that the QY (0.24%) of TTQF NPs is 2.2-fold than that of TTQF-SO_3_ (0.11%), TTQF-SO_3_ exhibited 14.7-fold higher uptake in J774A.1 cells than that of TTQF NPs at 120 min (Fig. 2d, Additional file 1: Fig. S5). This result indicated that TTQF-SO_3_ was easily absorbed by undifferentiated monocytes, which may be closely related to its surface charge.

Given the excellent macrophage uptake behavior of TTQF-SO_3_ in vitro, we further studied its biological distribution in vivo. As illustrated in Additional file 1: Fig. S8a, with 30 min after the injection of TTQF-SO_3_ (1.0 mg mL^−1^, 100 μL) through the tail vein, the vascular signal decreased significantly, while the liver signal increased remarkably, indicating that the probe was rapidly captured by the endothelial reticular system [29]. It is worth noting that the mouse tail lymph can be clearly observed at the time point of 2 h, indicating that TTQF-SO_3_ can enter the lymphatic system through blood circulation (Fig. 2e). This result can be attributed to its reasonable negative charge density, which enables it to pass through the interstitial matrix filled with collagen fibers and negatively charged glycosaminoglycans [30]. Furthermore, the lymph nodes and lymph vessel (LV) can be clearly observed after footpad injection of TTQF-SO_3_ at 60 min (Fig. 2f; Additional file 1: Fig. S8b). The result of cross-sectional signal profile of LVs displayed the LVs-to-muscle ratios of ∼ 8.9 with remarkably sharp features (the full width at half-maximums (FWHMs) of LVs was ∼ 130 μm). Overall, TTQF-SO_3_ could provide alternative modalities for imaging the lymphatic system intraoperatively.

**Fig. 3:**
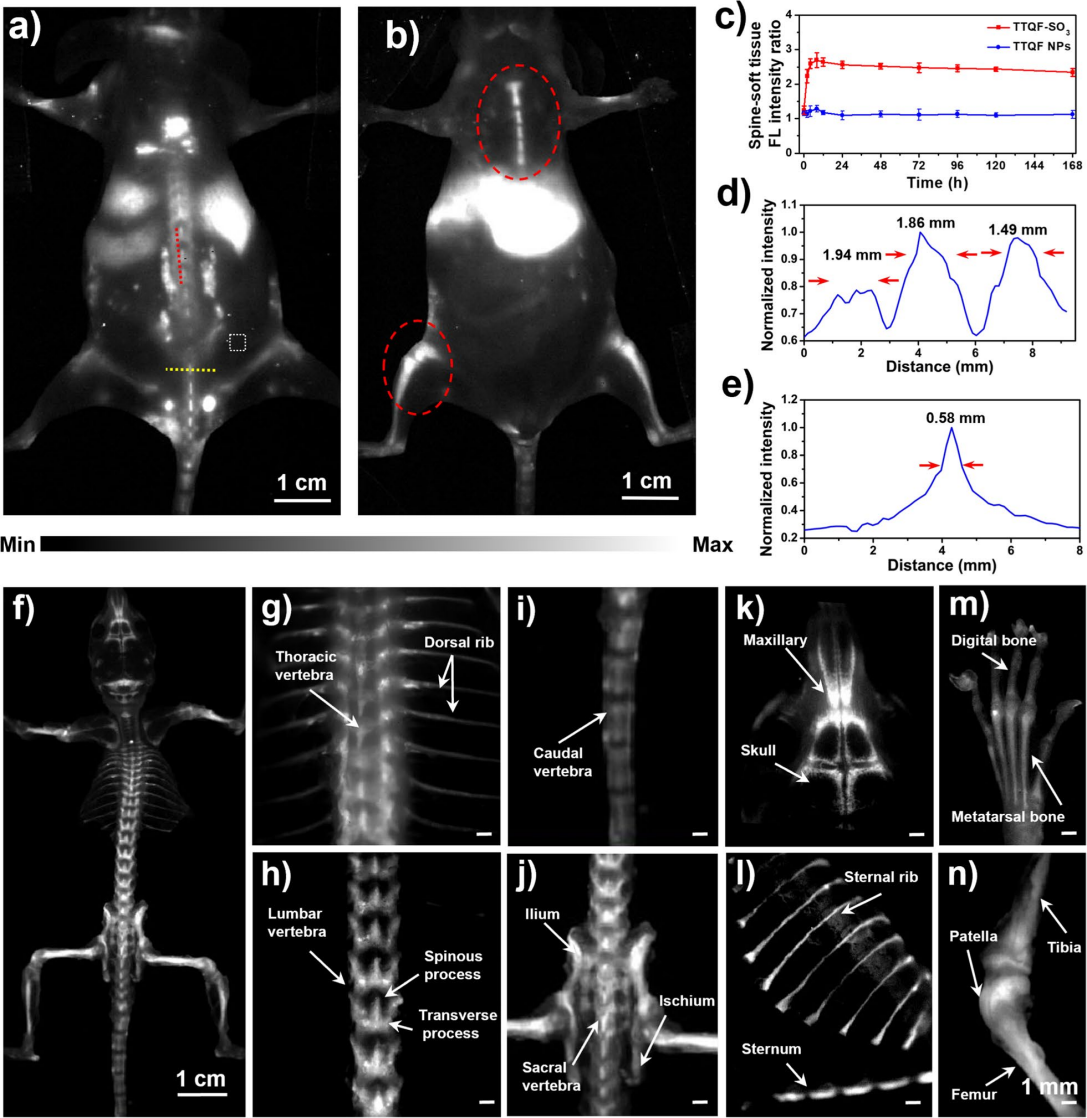
**a**, **b** NIR-II FI of BALB/c mice at 12 h post-injection using TTQF-SO_3_. White dotted box: soft tissue. Imaging parameters: 1300 filter, exposure 3000 ms. **c** Spine-soft tissue fluorescence ratio of TTQF-SO_3_ and TTQF NPs at different time-point. **d** FL intensity analysis of the red line in **a**. **e** FL intensity analysis of the yellow line in **a**. **f** NIR-II FI of the whole skeletal system using TTQF-SO_3_. **g**–**n** Stereomicroscope NIR-II FI of some pivotal skeletal system using TTQF-SO_3_. Imaging parameters: 1300 filter, exposure 500 ms. Error bars, mean ± SD (n = 3)

Before evaluation of bone-targeted imaging, the cytotoxicity of TTQF-SO_3_ was investigated. As demonstrated in Additional file 1: Fig. S9a, 3-(4,5-dimethyl thiazol-2-yl)-2,5-diphenyl tetrazolium bromide (MTT) assays showed that TTQF-SO_3_ showed very low cytotoxicity, and the survival rate of cells could reach more than 90% even at high concentration (0.2 mg mL^−1^). Hemolysis also showed that TTQF-SO_3_ had very low biotoxicity, even at 4 mg mL^−1^ concentration (Additional file 1: Fig. S9b). Next, TTQF-SO_3_ was intravenously injected (1.0 mg mL^−1^, 100 μL) into the tail of normal mice to measured its biodistribution using the 1300 nm LP filter. In addition, the non-functionalized precursor TTQF NPs was prepared as a control group by co-deposition with the amphiphilic Pluronic F-127. From NIR-II FI results (Fig. 3a, b; Additional file 1: Figs. S10 and S12), the TTQF-SO_3_ provided a high-sensitivity and signal-to-background ratios (SBRs) in the normal bones compared with TTQF NPs (Additional file 1: Figs. S11 and S13), revealing that the bones were easier to be recognized by a sulfonated fluorophore (TTQF-SO_3_) rather than an unsulfonated fluorophore (TTQF). The SBR of the spine was reached a peak of 2.7 at 8 h after injection with TTQF-SO_3_ (Fig. 3c). By contrast, the SBR was only reached a peak of 1.2 at 8 h after injection with TTQF NPs, which was far below than that of TTQF-SO_3_ at almost all time points (Fig. 3c). Furthermore, the above NIR-II FI results were quantitatively analyzed. As observed in Fig. 3d, parallel analysis (red line) along the spine showed three peaks of fluorescence intensity in the mouse injected with TTQF-SO_3_, high enough to distinguish different vertebrae. The FWHMs were ranging from 1.49 to 1.94 mm. Vertical analysis crossing the spine (yellow line) also exhibited a good differentiation (FWHM = 0.58 mm) between the vertebrae and others (Fig. 3e).

Then, the ex vivo NIR-II FI was performed on isolated bone tissues of the healthy mouse after injected with TTQF-SO_3_. As displayed in Fig. 3f, the entire skeletal system can be clearly imaged from the prone position after removing soft tissues. Moreover, the details of bone images at various sites were also performed using a NIR-II stereomicroscope (Fig. 3g–n). Figure 3g clearly displays the thoracic vertebrae and dorsal rib of the normal mouse. Figure 3h clearly exhibits the lumbar. It’s worth noting that the spinous and transverse processes can be observed in the images. Similarly, the caudal vertebrae can also be observed in Fig. 3i. In addition, Fig. 3j shows the ilium and ischium, and Fig. 3k displays the skull of the mouse. Likewise, the following images exhibits the sternum and sternal rib (Fig. 3l), digital and metatarsal bone (Fig. 3m), tibia, patella, and femur (Fig. 3n) in turn. It should be noted that the soft ribs of the chest were not shown in Fig. 3l, which means that TTQF-SO_3_ could not be enriched in the cartilage tissue on account of different composition of cartilage and bone tissue [31]. All the data suggested that the small-molecule TTQF-SO_3_ has specifically bone-binding affinity to successfully visualize the whole skeletal system in vivo, which has the potential for real-time intraoperative bone imaging.

**Fig. 4:**
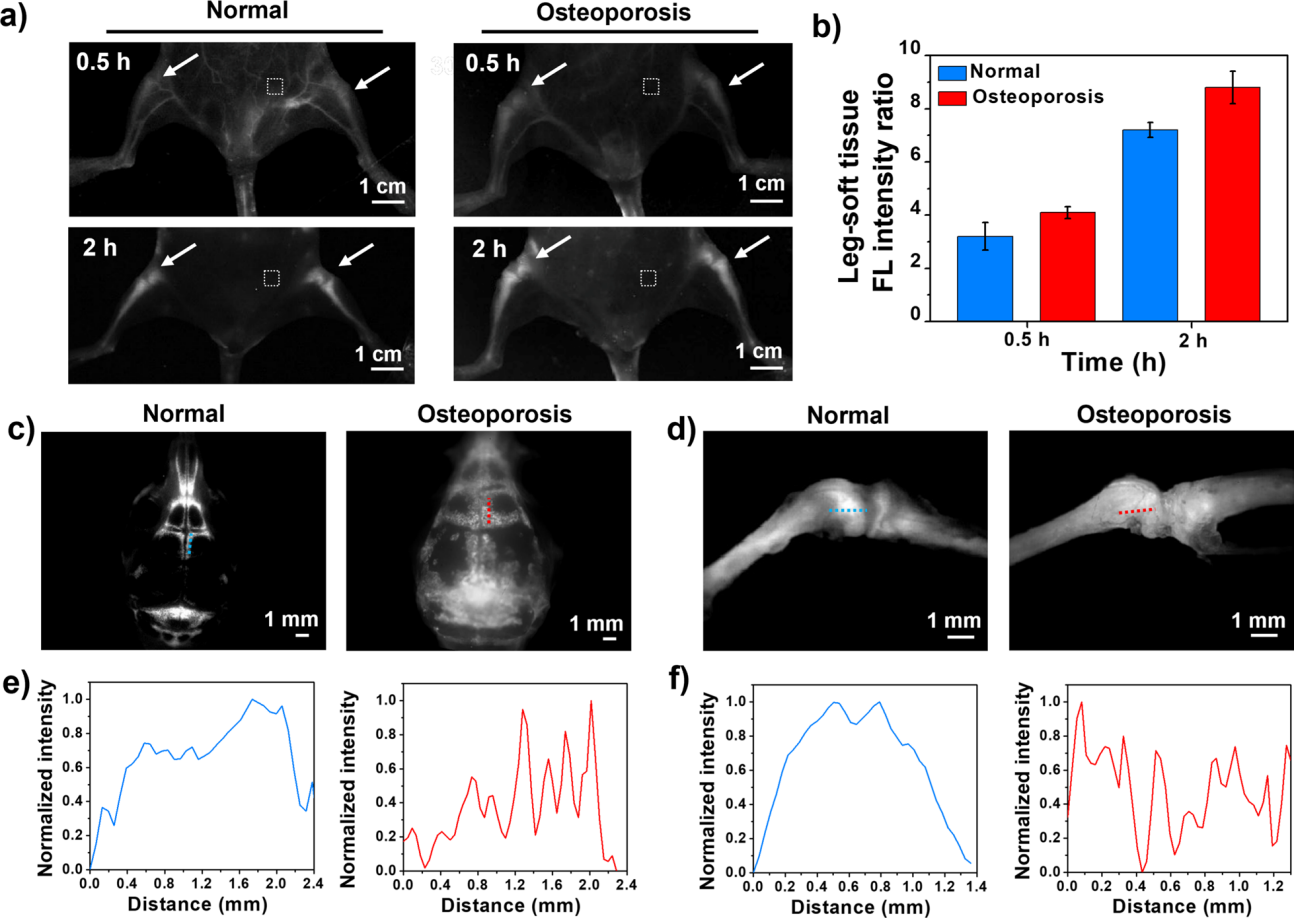
**a** NIR-II FI of normal mice and osteoporosis mice in supine position were obtained at 0.5 and 2 h after injection, respectively. Imaging parameters: 1300 filter, exposure 3000 ms. Dotted box in white: soft tissue. **b** Corresponding leg-tissue fluorescence signal ratio of the two groups. **c** NIR-II FI of skulls in normal (left) and osteoporotic mice (right). Imaging parameters: 1300 filter, exposure 500 ms. **d** NIR-II FI of shanks in normal (left) and osteoporotic mice (right). **e**, **f** Corresponding normalized FL intensity distribution along yellow/red lines in corresponding figures c and d. Error bars, mean ± SD (n = 3)

In view of the outstanding bone imaging quality of TTQF-SO_3_ in normal mice, osteoporosis model mice were selected for further evaluated its NIR-II bone-imaging. TTQF-SO_3_ (100 μL, 1.0 mg mL^−1^) was injected into normal mice (n = 3) and osteoporosis mice (n = 3) through the tail vein, and then NIR-II FI were taken at different time points (Additional file 1: Fig. S14). As exhibited in Fig. 4a, after 30 min injection, TTQF-SO_3_-based NIR-II FI exhibited significant differences between the legs of osteoporosis mice and normal mice. Within 2 h after injection, the SBR value in the legs of osteoporosis mice was higher than that of normal mice from beginning to end, and the quality of NIR-II FI was significantly improved (Fig. 4b). The higher accumulation of TTQF-SO_3_ in osteoporosis mice may be attributed to osteoclast activity. These osteoclast precursors belong to the monocyte/macrophage class and have similar phagocytosis capabilities, therefore, TTQF-SO_3_ can be easily accumulated into bone, which is consistent with cellular uptake data.

After removing the skin and viscera from osteoporotic mice, we further performed in vitro NIR-II FI analysis using an NIR-II microscope (Additional file 1: Fig. S15). Compared with normal mice, the osteoporotic mice had defects on the surface of their bones, particularly the skull and kneecaps (Fig. 4c, d). Further quantitative bone analysis in vitro showed that the fluorescence intensity curves of the skull and kneecap of normal mice showed a large broad peak, while those of osteoporosis mice exhibited many distinct sharp peaks (Fig. 4e, f). These results suggested that the bone surface of the osteoporosis mice was uneven. In addition, quantitative fluorescence signals of skulls and shanks ex vivo were further evaluated (Additional file 1: Fig. S16). The fluorescence intensity of TTQF-SO_3_ in skull and shank of osteoporotic mice increased by approximately 3.0- and 1.3-fold, respectively, higher than that of normal mice, indicating the superior accumulation ability for osteoporotic mice.

**Fig. 5:**
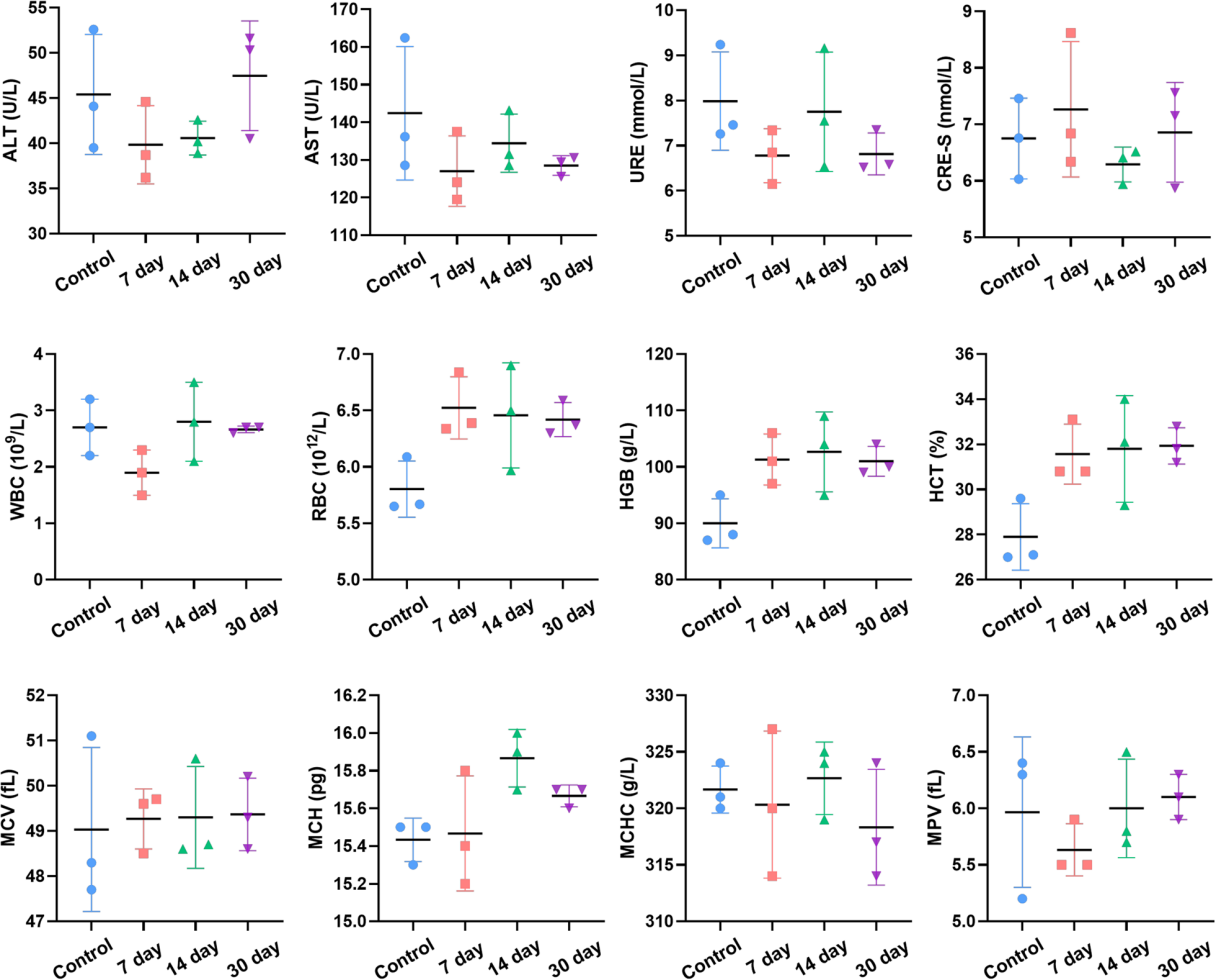
Blood routine tests of mice were performed after the treatment with TTQF-SO_3_ (100 μL, 1.0 mg mL^−1^) during 30 days. Error bars, mean ± SD (n = 3)

The in vivo biosafety of TTQF-SO_3_ was further evaluated. As shown in Figure S17 (Supporting Information), the main organs in vitro showed that TTQF-SO_3_ was mainly accumulated in liver and spleen. Moreover, no obvious inflammation or abnormalities were observed in major organs of mice after injection of TTQF-SO_3_, demonstrating that the fluorescent probe has good biocompatibility (Fig. 5; Additional file 1: Fig. S18). Furthermore, the normal mouse injected with TTQF-SO_3_ can still observe the fluorescence signals of the liver and bone after 21 days, indicating that the fluorophore can remain in vivo for a long time (Additional file 1: Fig. S19). Predictably, long-term retention feature of TTQF-SO_3_ in bone tissue may serve as targeting group to deliver drugs to primary bone tumors, bone metastases, osteoporosis, osteomyelitis and so on.

## Conclusion

In conclusion, an efficient bone targeting probe based on NIR-II fluorophore was successfully developed by grafting a specific number of sulfonate group. TTQF-SO_3_ shows excellent calcium-binding, bone-targeted features and outstanding biosecurity, which can be used for non-invasive and non-radiation bone tissues visualization and further monitoring of bone related diseases in vivo, such as osteoporosis. In vitro and in vivo results further support that our TTQF-SO_3_ has high imaging resolution and outstanding biocompatibility, which expected to be further applied in preclinical research of bone imaging. This study may become in vivo bone targeting design strategy besides the classical phosphate modification.

## Supplementary Information

**Additional file 1**: Experimental section and supporting figures associated with this article can be found in the online version.

## Data Availability

All data needed to support the conclusions are present in the paper and/or the Supporting Information. Additional data related to this study are available from the corresponding authors upon reasonable.
