# Supplementary material for: Quasi-dendritic sulfonate-based organic small molecule for high-quality NIR-II bone-targeted imaging
Source: J Nanobiotechnology. 2023 Jul 19;21:230. doi: 10.1186/s12951-023-01999-9 (PMC10354972; doi:10.1186/s12951-023-01999-9)
Supplement: Supplementary file 1 — Additional file 1: Experimental section and supporting figures associated with this article can be found in the online version. [file 12951_2023_1999_MOESM1_ESM.docx]

**Quasi-Dendritic Sulfonate-Based** **Organic Small Molecule for High-Quality NIR-II** **Bone-Targeted Imaging**

Pengfei Chen^a^, Fan Qu^b^, Liuliang He^a^, Mingfei Li^a^, Pengfei Sun^b^*, Quli Fan^b^, Chi Zhang^a^*, Daifeng Li^a^*

^a^ Department of Orthopedics, The First Affiliated Hospital of Zhengzhou University, Zhengzhou, 450052, China

E-mail: lidaifeng@zzu.edu.cn, zhangchi211@163.com

^b^ State Key Laboratory of Organic Electronics and Information Displays & Institute of Advanced Materials (IAM), Jiangsu Key Laboratory for Biosensors, Nanjing University of Posts & Telecommunications, Nanjing 210023, China

E-mail: iampfsun@njupt.edu.cn

**EXPERIMENTAL SECTION**

**Materials.** 4,9-Bis(5-bromo-thiophen-2-yl)-6,7-bis(4-(hexyloxy)phenyl)-[1,2,5]thiadiazolo[3,4-*g*] quinoxaline (**TTQ**, 97%), 2-(9,9-bis(6-bromohexyl)-9H-fluoren-2-yl)-4,4,5,5 -tetramethyl-[1,3,2]dioxaborolane (**FB**, 97%) were purchased from SunaTech Inc. 1, 3-propanesulfonate lactone was obtained from *J&K* Scientific Ltd. Unless indicated otherwise, all synthetic procedures were performed in an anhydrous and oxygenfree environment, and all reagents were received from commercial sources. These regents were used without further purification, except toluene which was dried and distilled with N_2_ before use. Cells were obtained from the Shanghai Laboratory Animal Center, Chinese Academy of Science (SLACCAS). Dulbecco’s Modified Eagle’s Medium (DMEM) and methyl thiazolyl tetrazolium (MTT) were obtained from KeyGen Biotech. Co., Ltd (Nanjing, China).

**Characterization.** The ^1^H NMR spectra were recorded with a Bruker Ultra Shield Plus 400 MHz spectrometer in deuterated dimethyl sulfoxide (DMSO-*d*_6_). The molecular analysis was performed on AutoflexIII Matrix-assisted laser desorption/ionization (MALDI) time-of-flight (TOF) mass spectrometry (Bruker Daltonics, USA). The morphology of nanoparticles was determined using a transmission electron microscope (HT7700, TEM) with an acceleration voltage of 100 KV. Dynamic light scattering (DLS) analysis were conducted on a commercial laser light scattering spectrometer (ALV-7004; ALV, Langen, Germany) equipped with a multi-τ digital time correlator and a He-Ne laser (at λ = 632.8 nm). The ⟨*R*h⟩ data were extracted through a CONTIN analysis. All samples we used for the test were optically cleared with Millipore filters (0.45 μm). The test was conducted at a 90° scattering angle and room temperature. A Shimadzu UV-3600 spectrophotometer was utilized to record the absorption spectra of our samples at room temperature. NIR-II fluorescence spectra were measured using an NIR-II spectrophotometer (Fluorolog 3, Horiba). NIR InGaAs was selected as the detector, with an excitation wavelength of 808 nm obtained from a diode laser operating at 25.0 ± 0.5 °C. After the raw emission data were collected, the fluorescence signal was further confirmed and corrected for the sensitivity of InGaAs detector profile and output through the T1c channel. The laser was purchased from Changchun New Industries Optoelectronics Technology Co., Ltd. The in vitro and in vivo NIR-II FI experiments were conducted on an NIR-II imaging system (Wuhan Grand-imaging Technology Co., Ltd) with various filters (1000-1100, 1100-1200, 1200-1300, and 1300-1400 nm) and two types of lenses (50 or 100 mm) under the 808 nm or 1064 nm laser irradiation. A 640 × 512 pixel two-dimensional InGaAs array from Princeton Instruments in NIR-II fluorescence windows was equipped in this NIR-II FI system. The MTT analysis was conducted using a PowerWave XS/XS2 microplate spectrophotometer (BioTek, Winooski, VT).

**Synthesis of TTQF-SO_3_:** The compound **TTQF-NH_2_** was synthesized from our previous report.^[1]^ Methanol (5 mL) was added to dissolve the compound **TTQF-NH_2_** (50 mg, 0.035 mmol). Then, 1, 3-propanesulfonate lactone (85 mg, 0.699 mmol) was dropwise added under N_2_. The reaction was stirred at 60 °C for 72 h. After cooling, the resulting product was evaporated and purified using column chromatography to afford the product **TTQF-SO_3_** (Yield: 41%). ^1^H NMR (400 MHz, DMSO-*d_6_*) *δ*: 0.67-0.75 (m, 8H), 0.98 (t, 8H), 1.11-1.13 (m, 16H), 1.36-1.45 (m, 18H), 1.45-1.59 (m, 6H), 1.85-193 (m, 6H), 2.09-2.13 (m, 16H), 2.32-2.36 (m, 16H), 2.42-2.56 (m, 16H), 2.32-2.36 (m, 16H), 2.57 (t, 8H), 4.10 (t, 4H), 7.05 (d, 4H), 7.38 (d, 6H), 7.66 (d, 2H), 7.75-7.84 (m, 8H), 7.95 (d, 4H), 9.16 (s, 2H). MALDI-TOF: calculated C_114_H_148_N_8_O_26_S_11_Na_7_^+^ (m/z): 2558, found 2557.

**NIR-II Fluorescence QY Test.** The QYs were determined according to the commercial reference dye IR-26, which has a QY value of 0.5% in 1,2-dichloroethane^[2]^. The parameter n is the refractive index of solvent. Five different concentrations around or less than an OD of 0.1 (approximately 0.1, 0.08, 0.06, 0.04 and 0.02) were measured, and all samples were analyzed at 25 °C. The sample and reference were excited by the same laser (808 nm). After comparing the slopes of the integrated fluorescence (emission at 1000-1500 nm), which was plotted against the absorbance for both the reference and samples, the QYs were calculated according to the following equation:


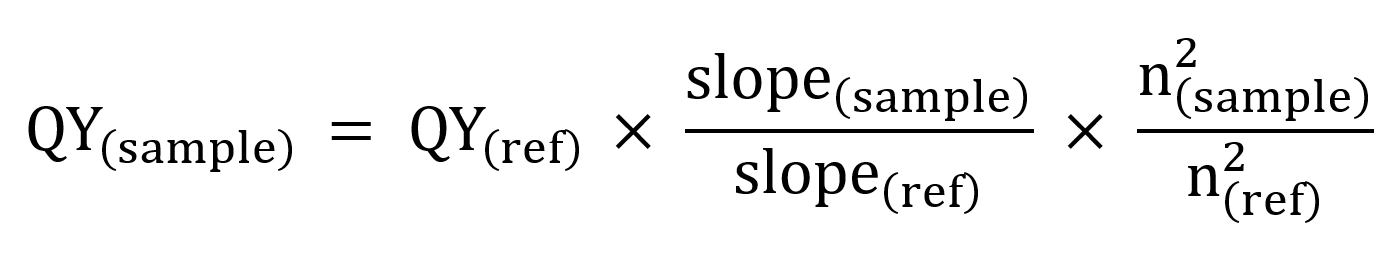


**Hemolysis Assay.** EDTA-stabilized blood samples obtained from healthy ICR mice were centrifuged (1200 rpm) for 8 min, followed by three washes with PBS. After the clarification of the supernatant, RBCs were diluted with PBS (v:v = 1:10) and 0.3 mL of RBC was added with (i) 1.7 mL of PBS (negative control), (ii) 1.7 mL of H_2_O (positive control) or (iii) 1.7 mL of the **TTQF-SO_3_** solution (4.0-0.25 mg mL^−1^). All NPs were incubated with RBCs at 37 °C for 4 h. Finally, the absorbance of each supernatant was measured at 541 nm and we further calculated the percentage of hemolyzed RBCs using the following formula (where *A*_sample_, *A*_negative_, and *A*_positive_ represent the absorbance values of **TTQF-SO_3_**, negative control, and positive control, respectively):


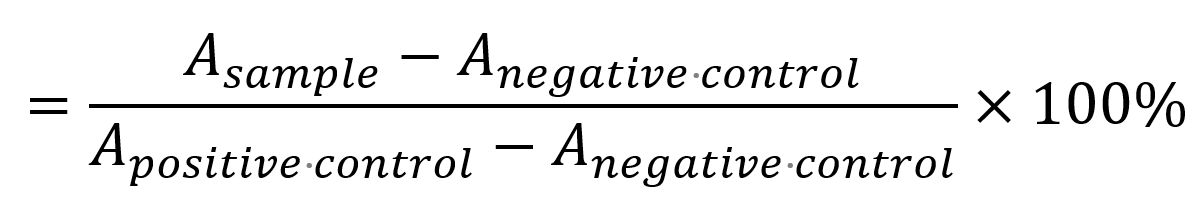


***In vitro* Cell Culture and Cellular Uptake.** The 3T3, 143B, and J774A.1 cells were used for the assessment of cellular uptake, respectively. First, cells, seeded 1 × 10^4^ per well, were incubated in DMEM in a 6-well plate at 37 °C and 5% CO_2_ condition for 12 h, and then incubated with **TTQF-SO_3_** and **TTQF NPs** (100 µM) for 30 min, 60 min, and 120 min, respectively. Afterward, the upper supernatant was sucked out and 1 mL of PBS was added to clean twice and remove dead cells. Subsequently, PBS was removed and 500 μL trypsin digestion solution with EDTA was added, and the cells were dissolved in an incubator at 37 °C and 5% CO_2_ for 1 min. Later 1 mL of DMEM was added and the cells were transferred into a 15 mL centrifuge tube and centrifuged for 3 min. The supernatant was then removed, and 100 μL of PBS was added to the centrifuge tube. Finally, the cells were transferred to a 96-well plate, where NIR-II fluorescence images of the two groups were captured under the 808 nm laser excitation.

***In Vitro* Cytotoxicity Assay.** The 3T3, 143B, and J774A.1 cells were cultured with DMEM supplemented with 10% FBS. The surrounding environment is at 37 ℃ with a humidified 5% CO_2_. Cells (1×10^4^ cells/well) were incubated in a 96-well plate for 24 h, then the medium was substituted with 100 µL of fresh DMEM containing **TTQF-SO_3_** with concentrations of 0, 6.25, 12.5, 25, 50, 100, and 200 µg/mL, respectively. Cells were cultured with **TTQF-SO_3_** for another 24 h. After that, each well of the microliter plate was added with 10 μL of 3-(4,5-dimethyl thiazol-2-yl)-2,5-diphenyl tetrazolium bromide (MTT) solution and the plate was cultured for accessional 2 h in CO_2_ incubator. Finally, use the Bio-tek Synergy HTX microplate spectrophotometer to determine the 450 nm absorbance of each well. The following formula was used to calculate the viability of cell growth: Viability (%) = (mean absorbance value of treatment group/mean absorbance value of control group) × 100%.

**Animal Experiments.** The animal protocols used in this study were approved by the Institutional Committee on the Ethics of Animal Experiments of Zhengzhou University, Zhengzhou, China. All animal procedures were performed in compliance with the Guide for the Care and Use of Laboratory Animals from the National Institutes of Health.

**Preparation of Osteoporosis Model.** 8-week-old female BALB/c mice were anesthetized with 10% chloral hydrate and fixed on the operating table. Shave the hair on both sides of the waist and back, disinfect with iodine, open the mouth to remove both ovaries, sew and disinfect. After surgery, the animals were kept in separate cages until they naturally woke up. The mice developed osteoporosis after four weeks of feeding.

***In Vivo* NIR-II Fluorescence Imaging.** 8-week-old female BALB/c normal mice and osteoporosis mice (n = 3/ per group) were intravenously injected with **TTQF-SO_3_** (1.0 mg/mL, 100 μL), respectively. The mice were imaged alive by anesthetizing them with isoflurane during the test time (just about 5 min) in the case of the eﬀect of respiration. The real-time *in vivo* NIR-II fluorescence imaging was performed at diﬀerent post-injection times by using an *in vivo* NIR-II fluorescence imaging system. The analysis of the signal intensity of NIR-II image was performed using the NIR-II *in vivo* imaging system software.

**SNR Analysis.** The imaging SNR was calculated according to the following equation:

***SNR* = *I*_s_/*I*_n_**

The parameters ***I*_s_** and ***I*_n_** represent the fluorescence signal of the interested region and the noise signal of normal tissue far away from the region of interest, respectively.

**Pathology Analysis.** All major organs (heart, liver, spleen, lung, and kidney) were achieved from healthy ICR mice (n = 3 per group). After injection of **TTQF-SO_3_** (1.0 mg/mL, 100 μL) at 7 days, they were embedded in an optimal cutting temperature sample (Tissue-Tek, Sakura Finetek, USA). After that, these samples were sliced into 4 μm sections with a microtome in the cryostat at -20 °C and then transferred to a microscope slide for analysis of hematoxylin-eosin (H&E).

**Blood Testing.** 8-week-old female BALB/c mice were divided into 4 groups with 3 mice in each group, totaling 12 mice. The first group did not do any treatment, and the second to fourth groups injected nanoparticles (1.0 mg/mL, 100 µL) through the tail vein. The first group did not undergo any treatment, while the 2-4 groups took blood samples for analysis and determination of liver function markers (alanine aminotransferase (ALT), aspartate aminotransferase (AST)) and renal function indicators (creatine (CRE), urea (URE)) at 7, 14, and 30 days, respectively; There are also blood routine tests including white blood cells (WBC), lymphocytes (LYM), hematocrit (HCT), hemoglobin (HGB), red blood cells (RBC), red blood cell distribution width (RDW), red blood cell hemoglobin concentration (CHC), mean platelet volume (MPV), HGB (heme), MCHC (mean heme concentration), and eosinophilic white blood cells (EOS).

**Statistical Analysis.** Graphpad Prism was used for statistical analysis. Quantitative data were performed for at least three times and the data were expressed as mean± standard deviation (SD). Data were analyzed for statistical significance using Student’s t-test. *P < 0.05 was considered statistically significant, while **P < 0.01 and ***P < 0.001 were considered highly and extremely significant.

**
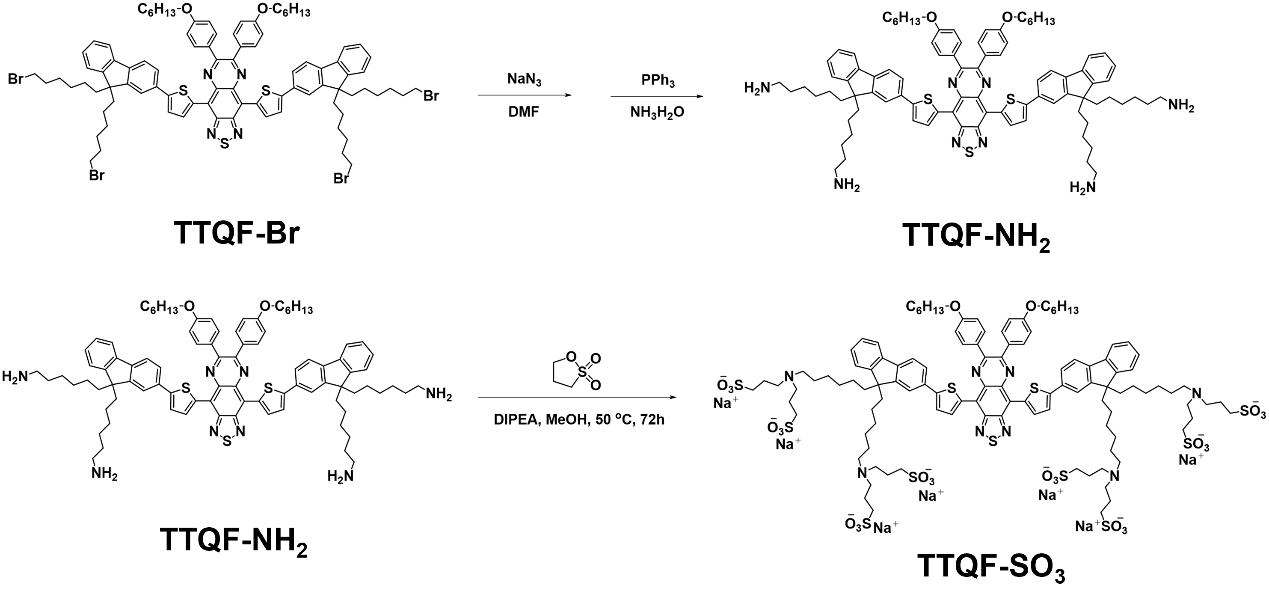
**

**Scheme S1.** The synthetic routes of **TTQF-SO_3_**.


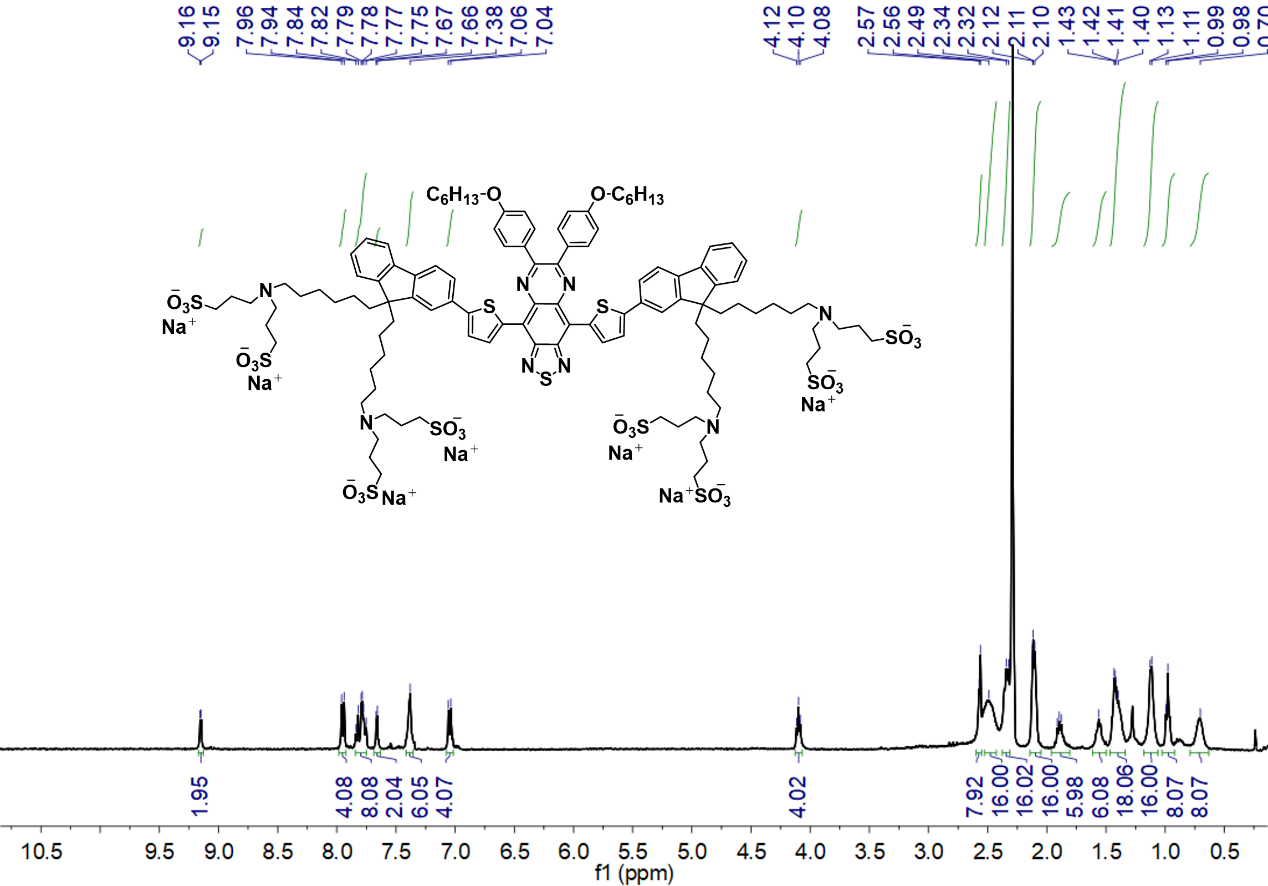


**Figure S1.** ^1^H NMR spectrum of **TTQF-SO_3_** in DMSO-*d_6_*.


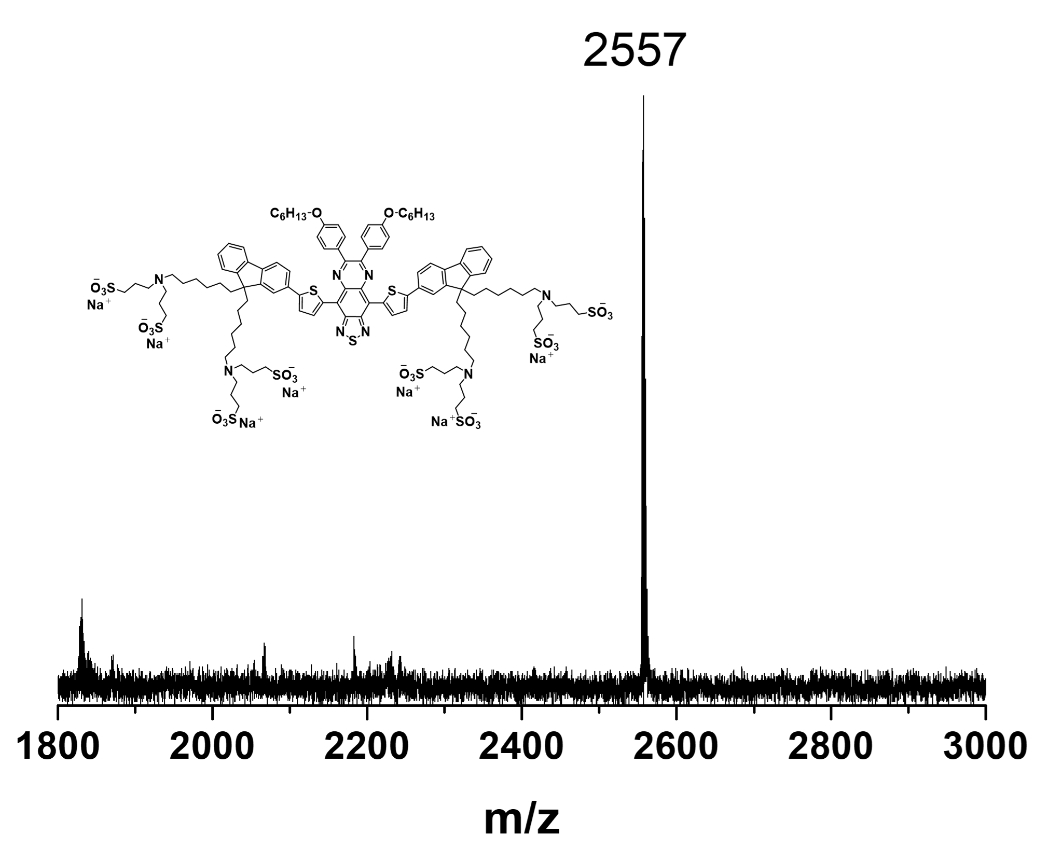


**Figure S2.** MALDI-TOF mass spectrometry of **TTQF-SO_3_**.


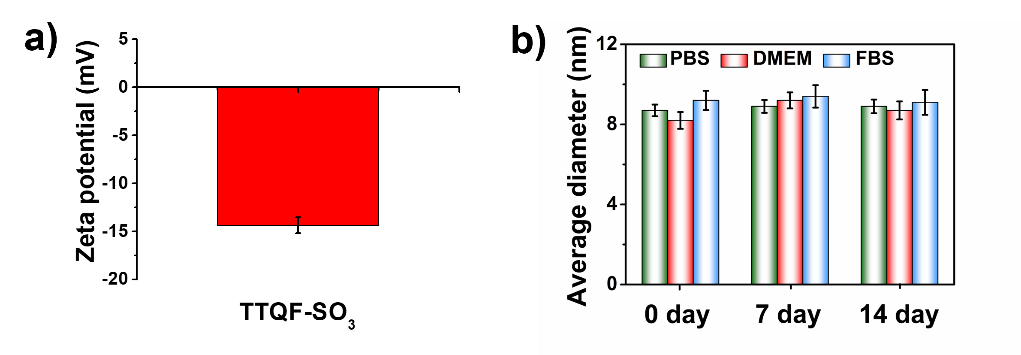


**Figure S3.** a) Zeta potential of **TTQF-SO_3_** in PBS (n = 5). b) Average diameters of **TTQF-SO_3_** in PBS, DMEM, or FBS for different time periods (n = 3).


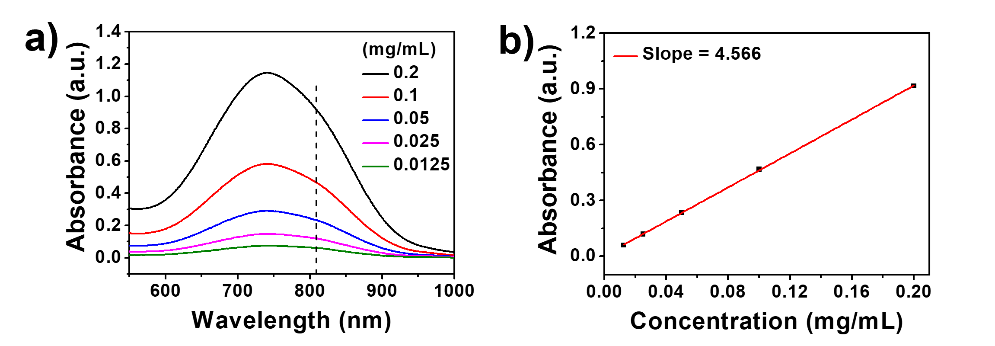


**Figure S4.** The molar extinction coefficient of **TTQF-SO_3_** at 808 nm in water.


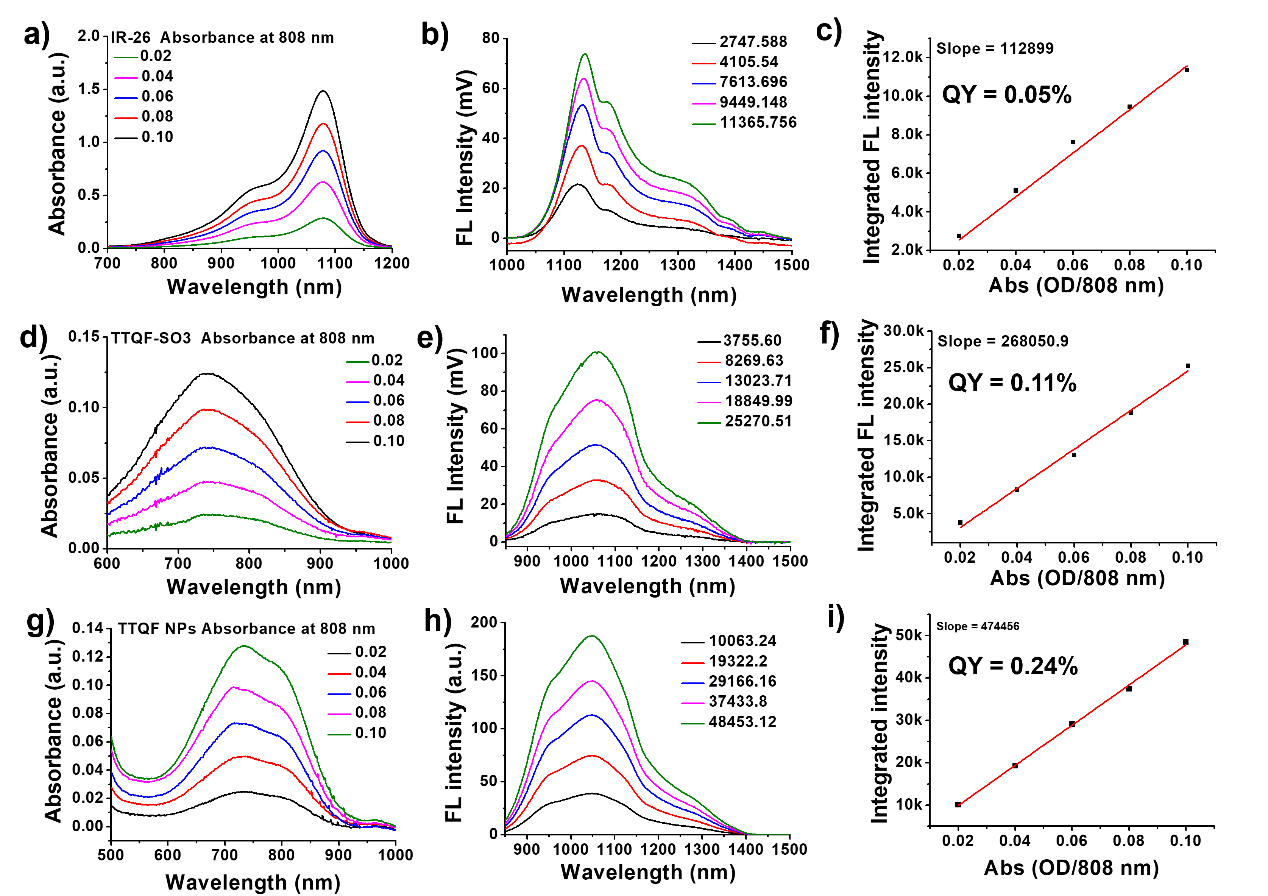


**Figure S5.** Quantum yield measurements of **TTQF-SO_3_** and **TTQF NPs** in different states with IR-26 as the reference sample. a-c) Absorption and emission spectra and relatively quantified analysis of IR-26 in DCE. d-f) Absorption and emission spectra and relatively quantified analysis of **TTQF-SO_3_** in water. g-i) Absorption and emission spectra and relatively quantified analysis of **TTQF NPs** in water.


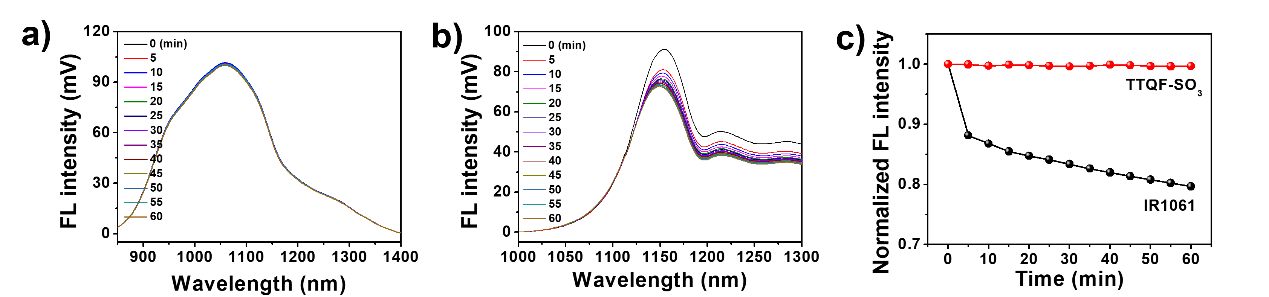


**Figure S6.** a-b) Fluorescence emission spectra recorded for **TTQF-SO_3_** and IR1061 in DMSO after exposed to continuous illumination at 808 nm (0.33 W cm^−2^) for 60 min, respectively. c) Corresponding normalized fluorescence intensity of dyes solution.


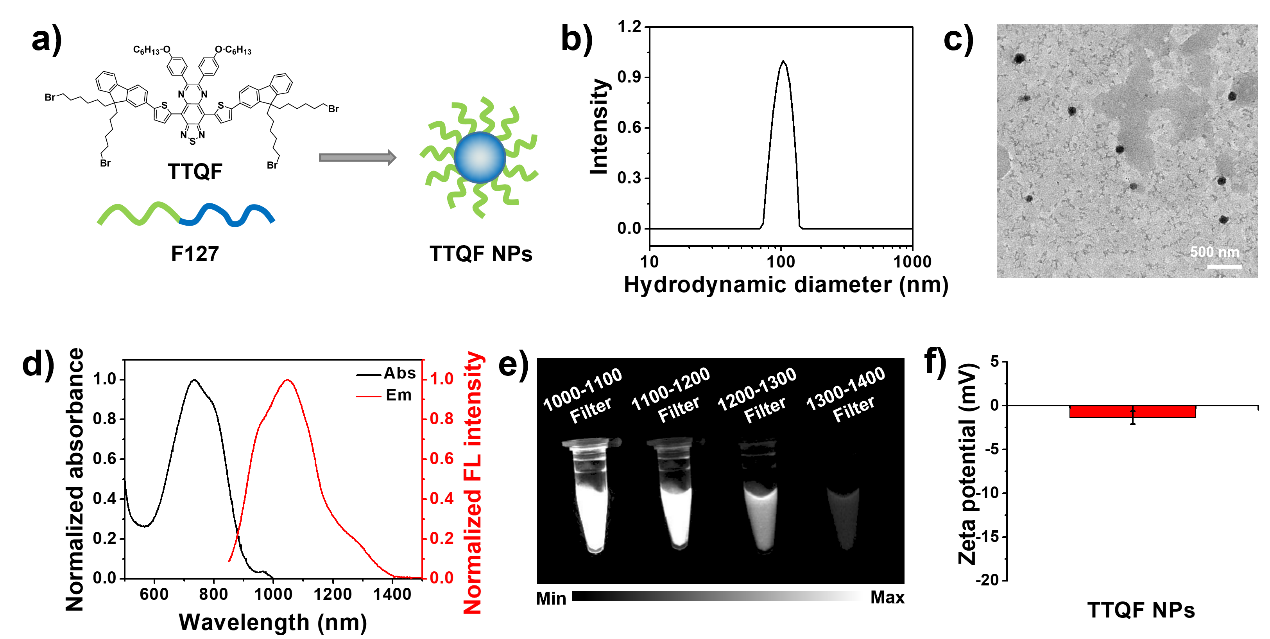


**Figure S7.** a) Schematic diagram of TTQF NPs preparation. b) and c) DLS and TEM image of TTQF NPs in water. d) Normalized absorbance and emission spectra of TTQF NPs in water. e) NIR-II signals of TTQF NPs obtained with different filters at the same concentration (0.1 mg mL^−1^) in water. f) Zeta potential of TTQF NPs in water (n = 5).


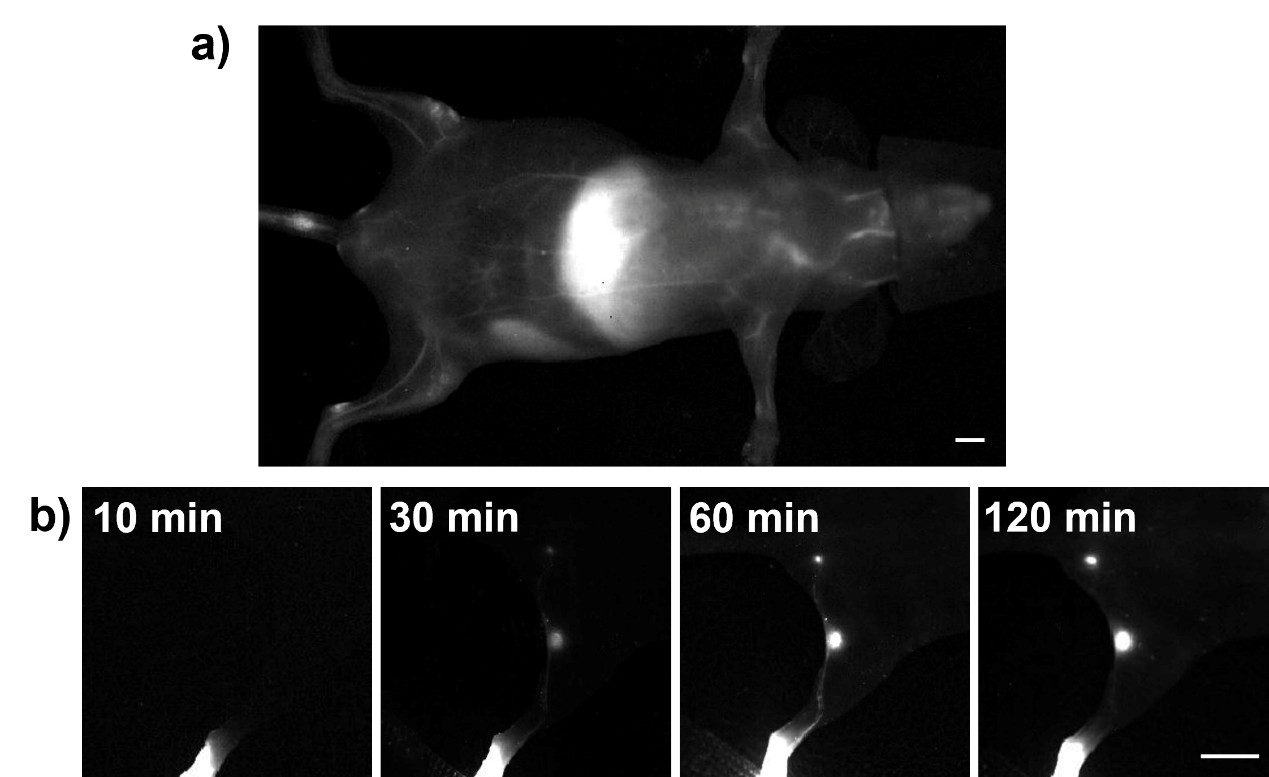


**Figure S8.** a) NIR-II FI of **TTQF-SO_3_** in mice after intravenous injection. b) NIR-II FI of lymph nodes in mouse leg at different time points after footpad injection of **TTQF-SO_3_**. Imaging parameters: 1300 filter, 3000 ms exposure time. Scale bar, 1.0 cm.


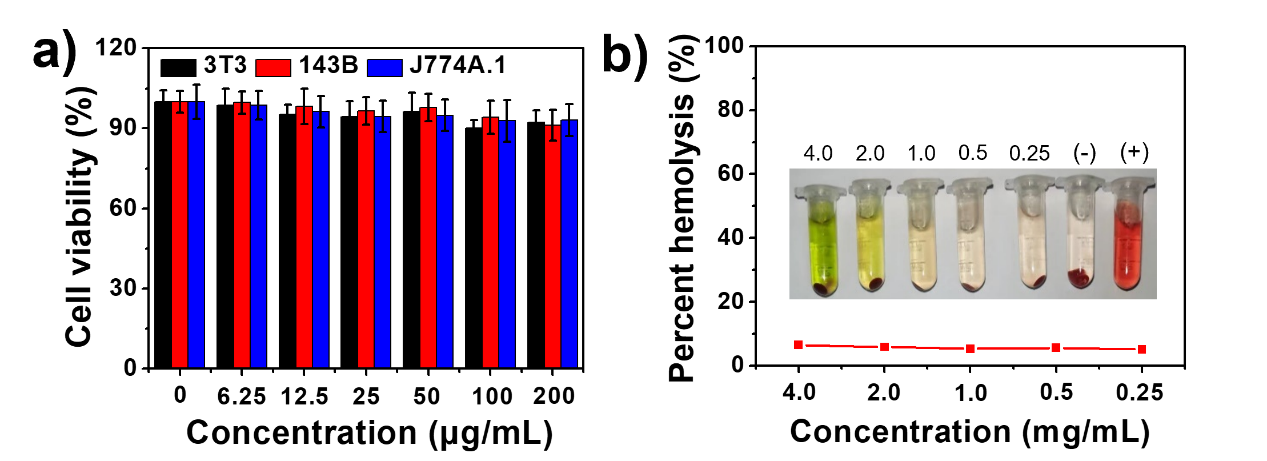


**Figure S9.** a) Relative viability of 3T3, 143B, and J774A.1 cells after treatment with **TTQF-SO_3_** at different concentrations. b) The hemocompatibility of **TTQF-SO_3_**. Hemolytic percent of RBCs treated with **TTQF-SO_3_** at various concentrations ranging from 4.0 to 0.25 mg mL^‒1^ after 4 h incubation at room temperature.


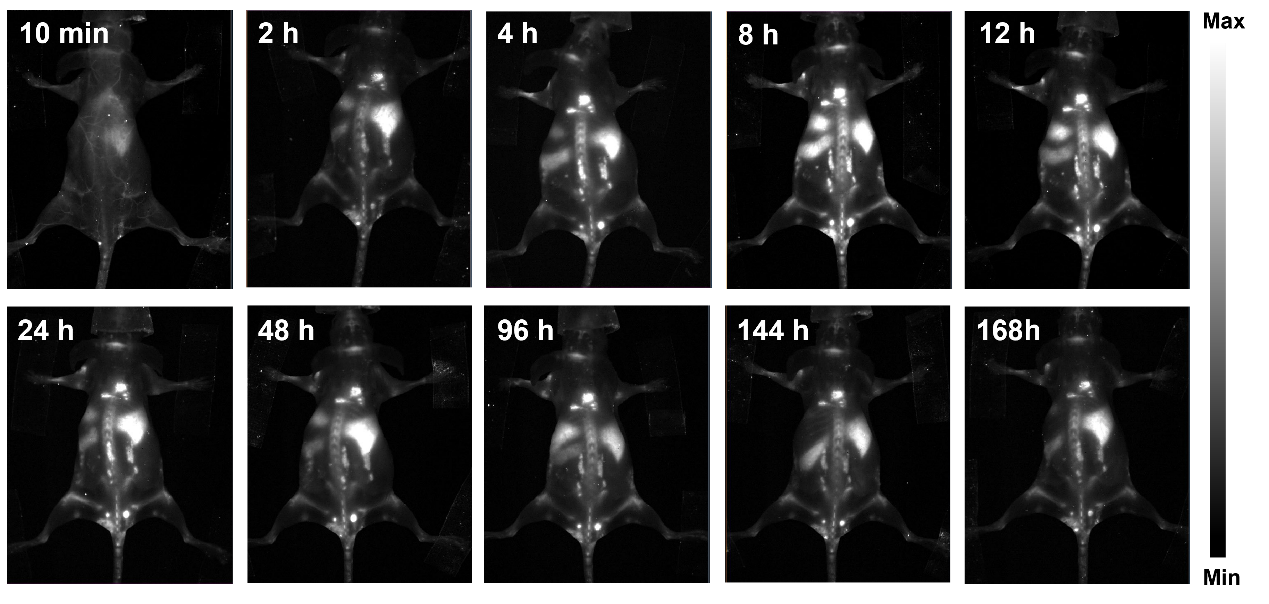


**Figure S10.** Real-time NIR-II FI of BALB/c mice (n=3) by **TTQF-SO_3_** with images in prone position acquired at 10 min, 2 h, 4 h, 8 h, 12 h, 24 h, 48 h, 96 h, 144 h, and 168 h post-injection, respectively. Imaging parameters: 1300 filter, 3000 ms exposure time. Scale bar, 1.0 cm.


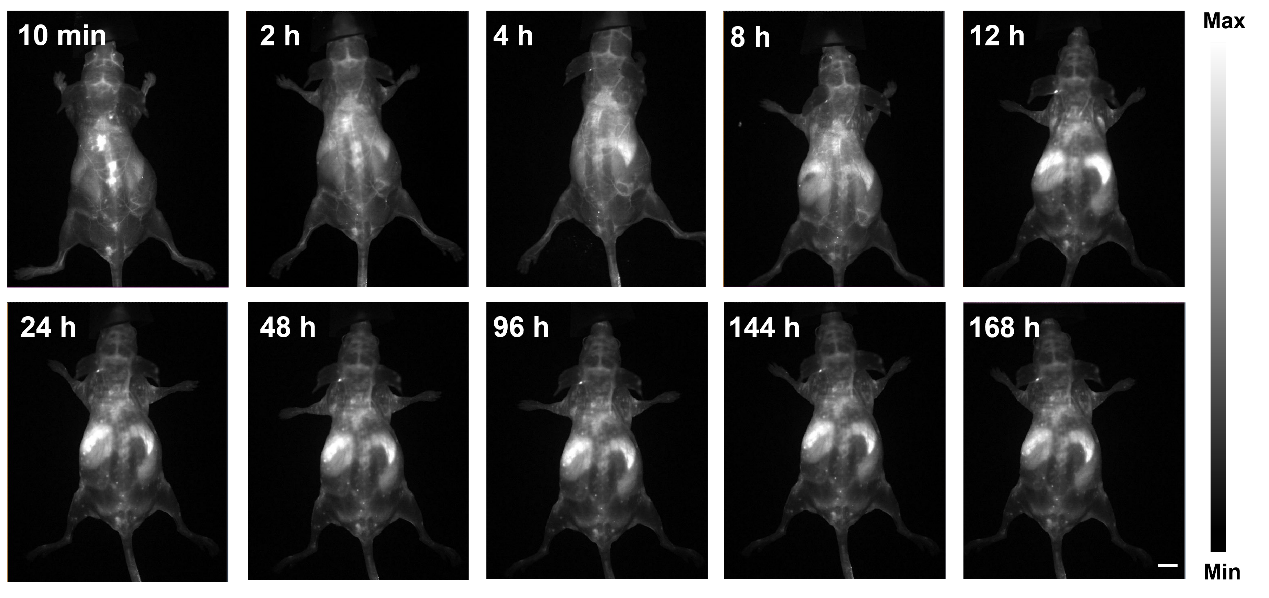


**Figure S11.** Real-time NIR-II FI of BALB/c mice (n = 3) by **TTQF NPs** with images in prone position acquired at 10 min, 2 h, 4 h, 8 h, 12 h, 24 h, 48 h, 96 h, 144 h, and 168 h post-injection, respectively. Imaging parameters: 1300 filter, 3000 ms exposure time. Scale bar, 1.0 cm.


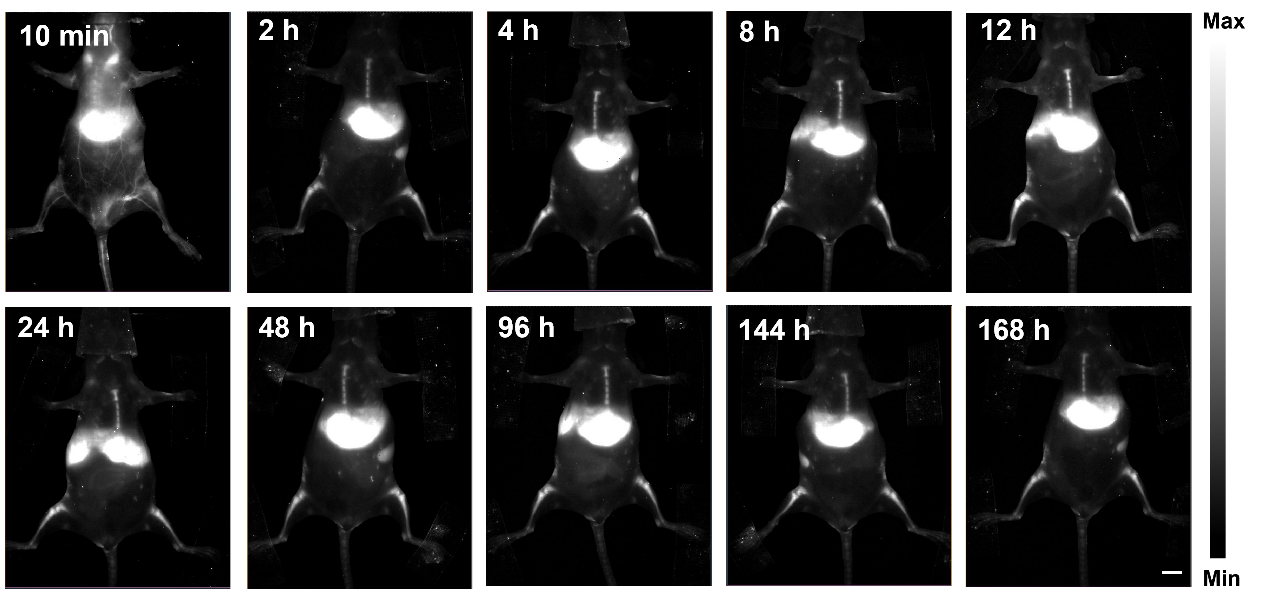


**Figure S12.** Real-time NIR-II FI of BALB/c mice (n = 3) by **TTQF-SO_3_** with images in supine position acquired at 10 min, 2 h, 4 h, 8 h, 12 h, 24 h, 48 h, 96 h, 144 h, and 168 h post-injection, respectively. Imaging parameters: 1300 filter, 3000 ms exposure time. Scale bar, 1.0 cm.


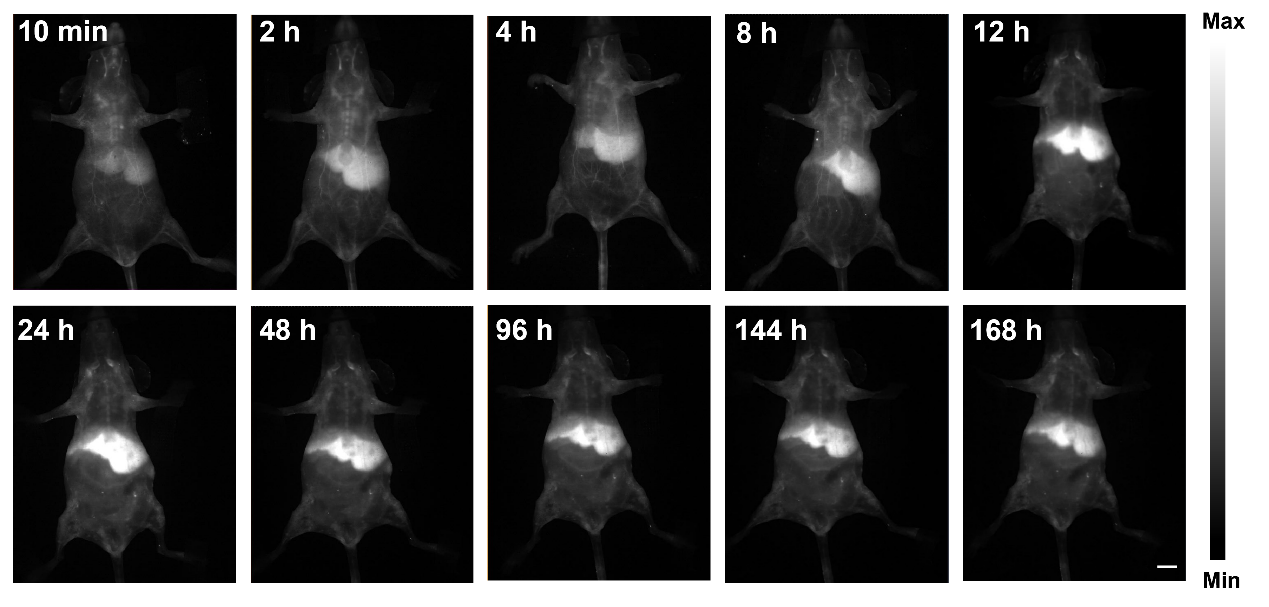


**Figure S13.** Real-time NIR-II FI of BALB/c mice (n = 3) by **TTQF NPs** with images in supine position acquired at 10 min, 2 h, 4 h, 8 h, 12 h, 24 h, 48 h, 96 h, 144 h, and 168 h post-injection, respectively. Imaging parameters: 1300 filter, 3000 ms exposure time. Scale bar, 1.0 cm.


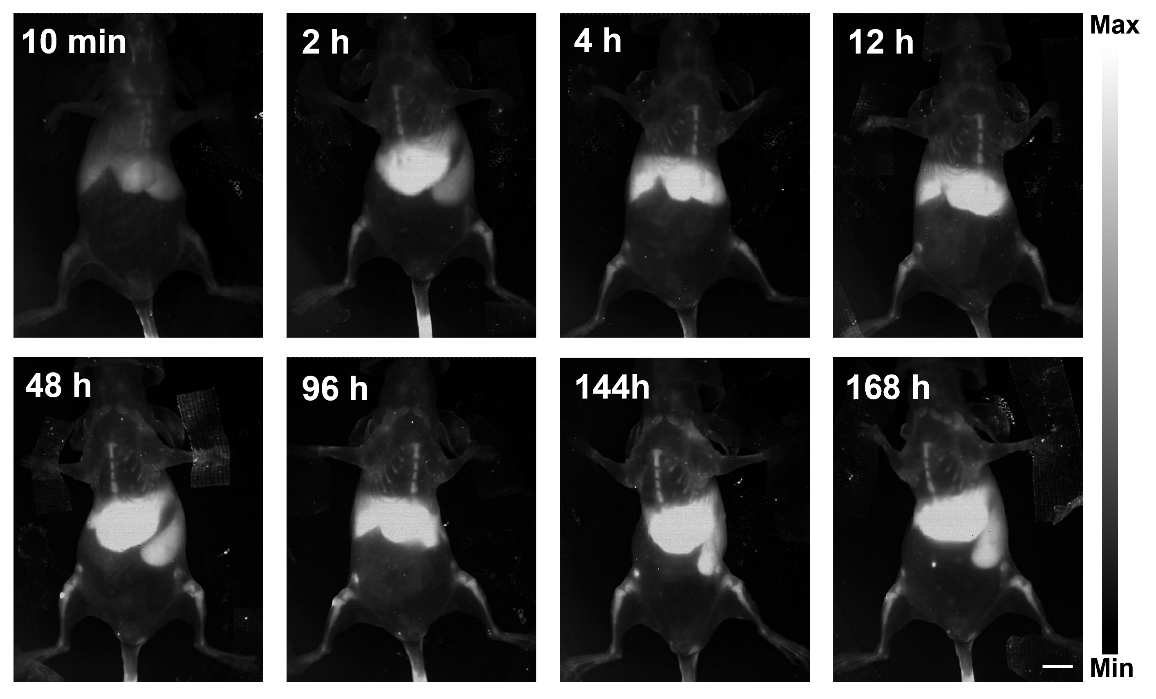


**Figure S14.** Real-time NIR-II FI of osteoporosis BALB/c mice (n = 3) by **TTQF-SO_3_** with images in supine position acquired at 10 min, 2 h, 4 h, 12 h, 48 h, 96 h, 144 h, and 168 h post-injection, respectively. Imaging parameters: 1300 filter, 3000 ms exposure time. Scale bar, 1.0 cm.


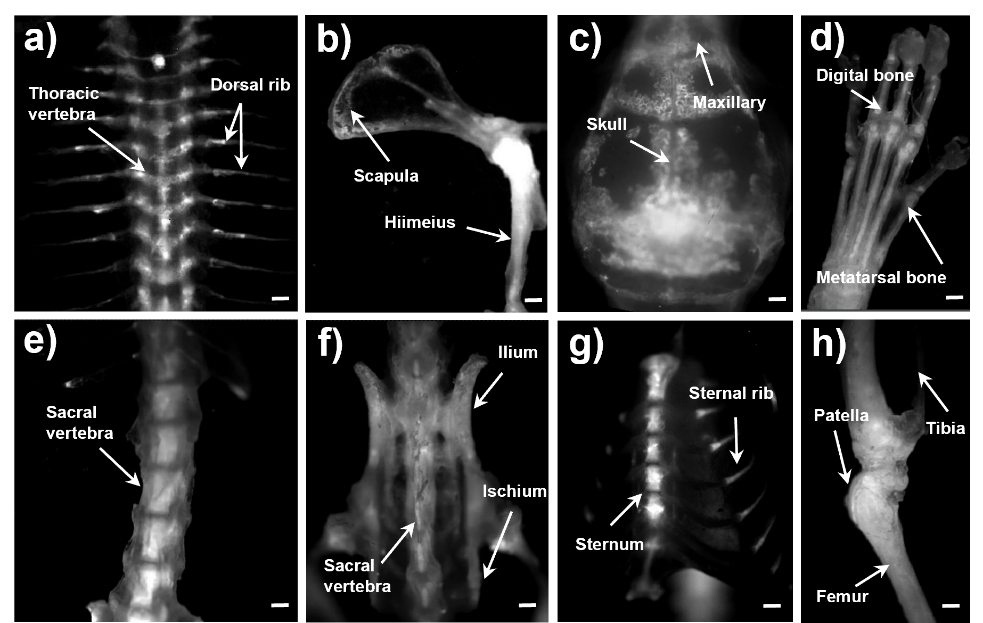


**Figure S15.** Ex vivo NIR-II imaging of the isolated bone tissues of the osteoporotic mouse. a-h) Zoom-stereo NIR-II FI of thoracic vertebra and dorsal rib, scapula and hiimeius, skull, metatarsal bone and digital bone, sacral vertebra, ilium and sacral vertebra, sternum and sternal rib, patella, femur and tibia, respectively. Scale bar, 1.0 mm. Imaging parameters: 1300 filter, 500 ms exposure time.


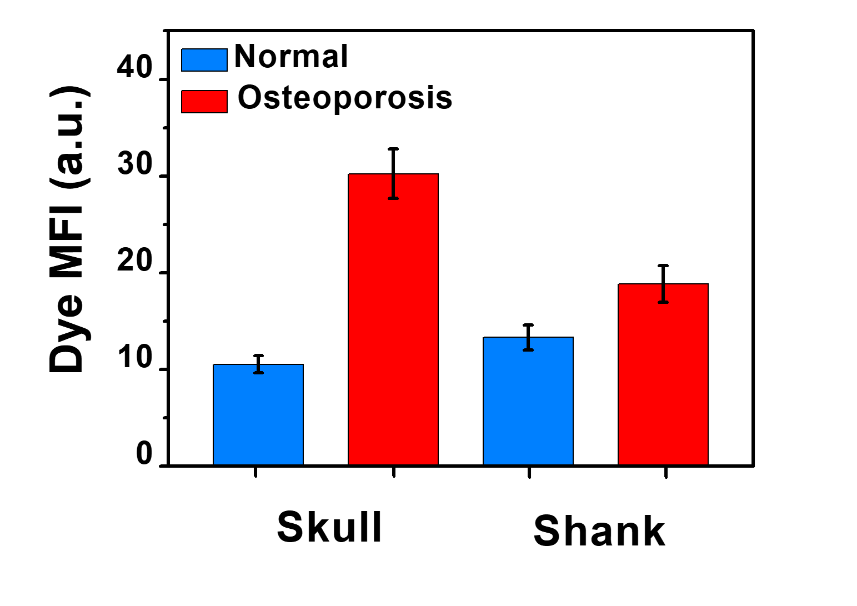


**Figure S16.** Quantitative fluorescence signals of skulls and shanks ex vivo in normal and osteoporotic mice.


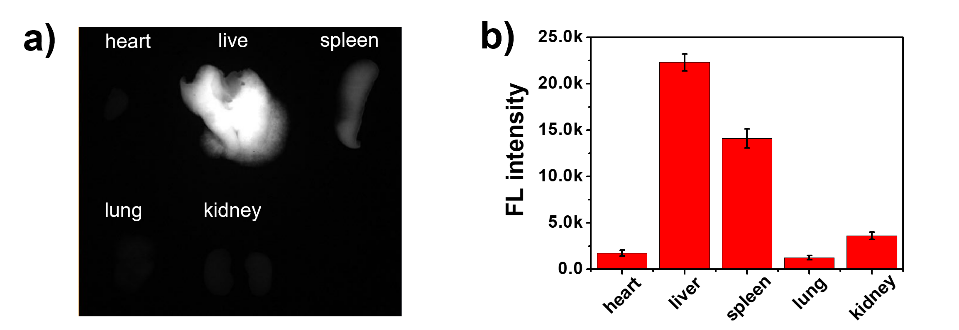


**Figure S17.** a) Ex vivo NIR-II FI of main organs of **TTQF-SO_3_** after 48 h intravenous administration. b) corresponding signal quantification (n = 3). Imaging parameters: 1300 filter, 500 ms exposure time.


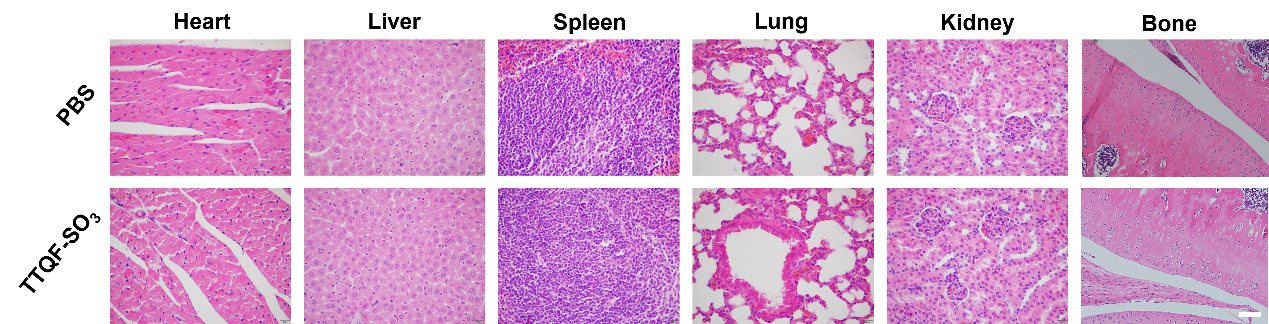


**Figure S18.** Images of H&E-stained main mouse organs and bone tissue. Scale bars, 100 µm.


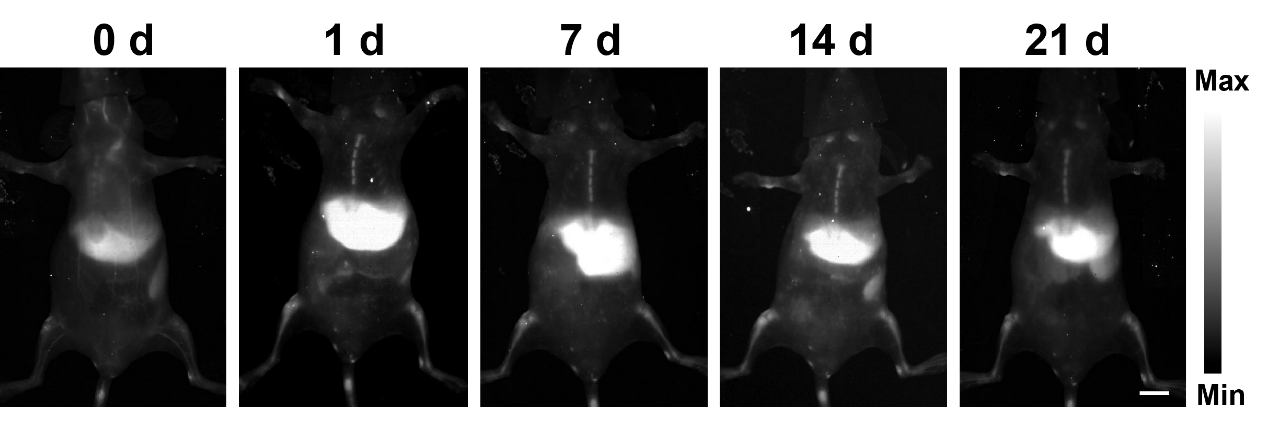


**Figure S19.** NIR-II FI of the normal mouse at different time points after injected with **TTQF-SO_3_**. Imaging parameters: 1300 filter, 3000 ms exposure time. Scale bars, 1.0 cm.

**Table S1**. Details of imaging parameters were used in each image of this work.

| Figures | Dyes | Laser (nm/mWcm^-2^) | Filter sets | Exposure time (ms) |
| --- | --- | --- | --- | --- |
| Fig. 1e | TTQF-SO_3_  (0.1 mg/mL) | 808/220 | 1000-1100  1100-1200  1200-1300  1300-1400 | 100  100  100  100 |
| Fig. 2a, 2c | TTQF-SO_3_  (0.1 mg/mL) | 808/220 | 980 | 50 |
| Fig. 2e | TTQF-SO_3_  (0.1 mg/mL) | 808/220 | 1300 | 3000 |
| Fig. 3a-b | TTQF-SO_3_  (1.0 mg/mL) | 808/220 | 1300 | 3000 |
| Fig. 3f-n | TTQF-SO_3_  (1.0 mg/mL) | 808/220 | 1300 | 500 |
| Fig. 4a | TTQF-SO_3_  (1.0 mg/mL) | 808/220 | 1300 | 3000 |
| Fig. 4c-d | TTQF-SO_3_  (1.0 mg/mL) | 808/220 | 1300 | 500 |

**References**

[1] S. Chen, H. Miao, X. Jiang, P. Sun, Q. Fan, W. Huang, Starlike polymer brush-based ultrasmall nanoparticles with simultaneously improved NIR-II fluorescence and blood circulation for efficient orthotopic glioblastoma imaging. *Biomaterials* **2021**, *275*, 120916.

[2] E. D. Cosco, J. R. Caram, O. T. Bruns, D. Franke, R. A. Day, E. P. Farr, M. G. Bawendi, E. M. Sletten, Flavylium polymethine fluorophores for near- and shortwave infrared imaging. *Angew. Chem. Int. Ed.* **2017**, *56*, 13126-13129.
